# Supplementary figures and images for: Enhancing pathogen identification through AI-assisted metagenomic sequencing
Source: Front Microbiol. 2025 Sep 19;16:1634194. doi: 10.3389/fmicb.2025.1634194 (PMC12493982; doi:10.3389/fmicb.2025.1634194)

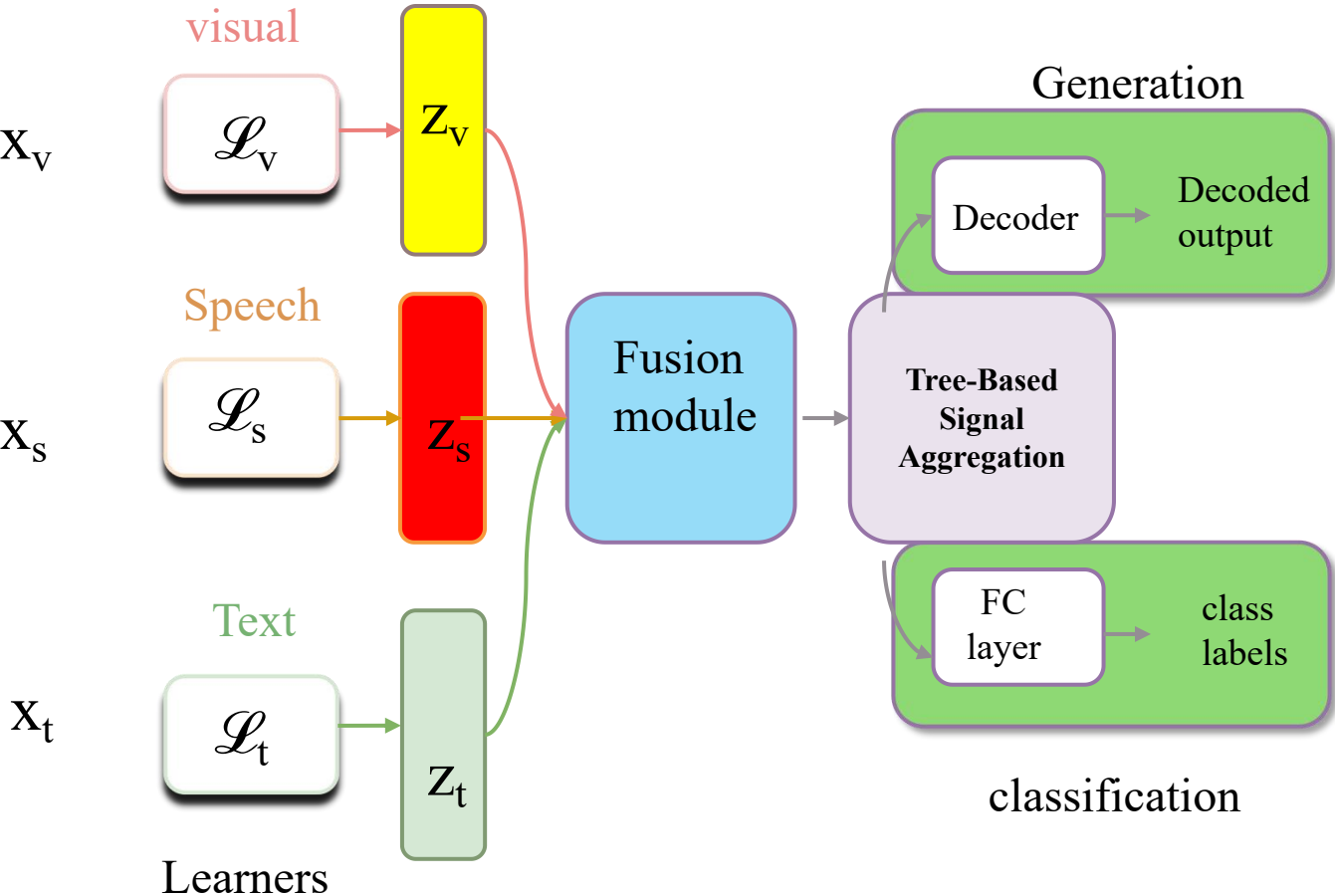

Supplement: Supplementary file 1 [file Data_Sheet_1.pdf]

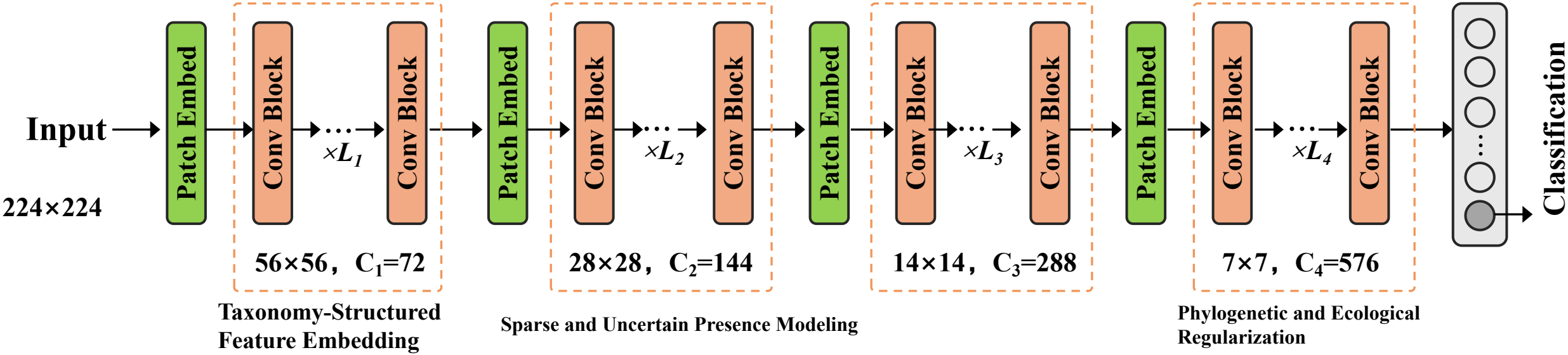

Supplement: Supplementary file 2 [file Data_Sheet_2.pdf]

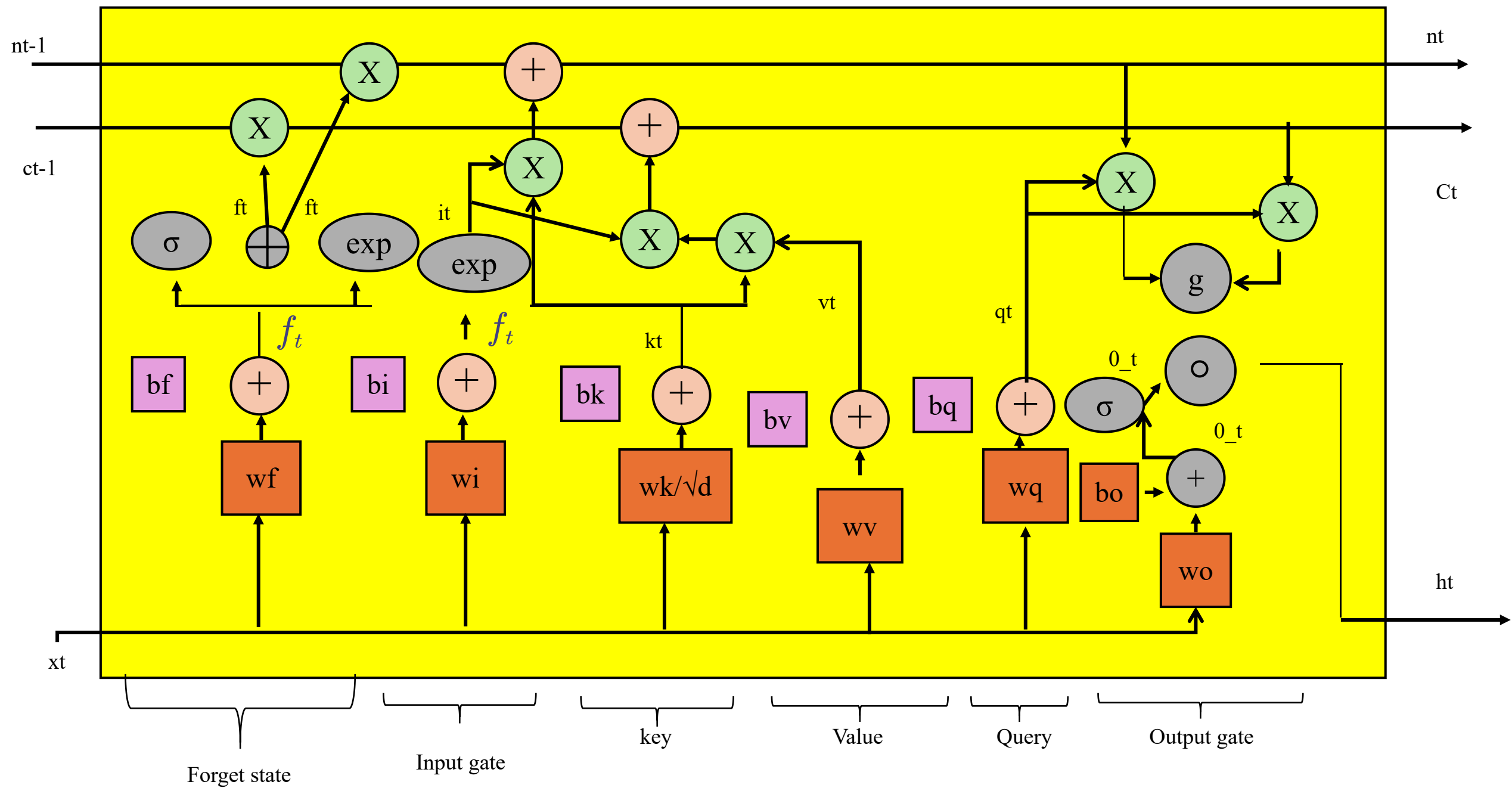

Supplement: Supplementary file 3 [file Data_Sheet_3.pdf]

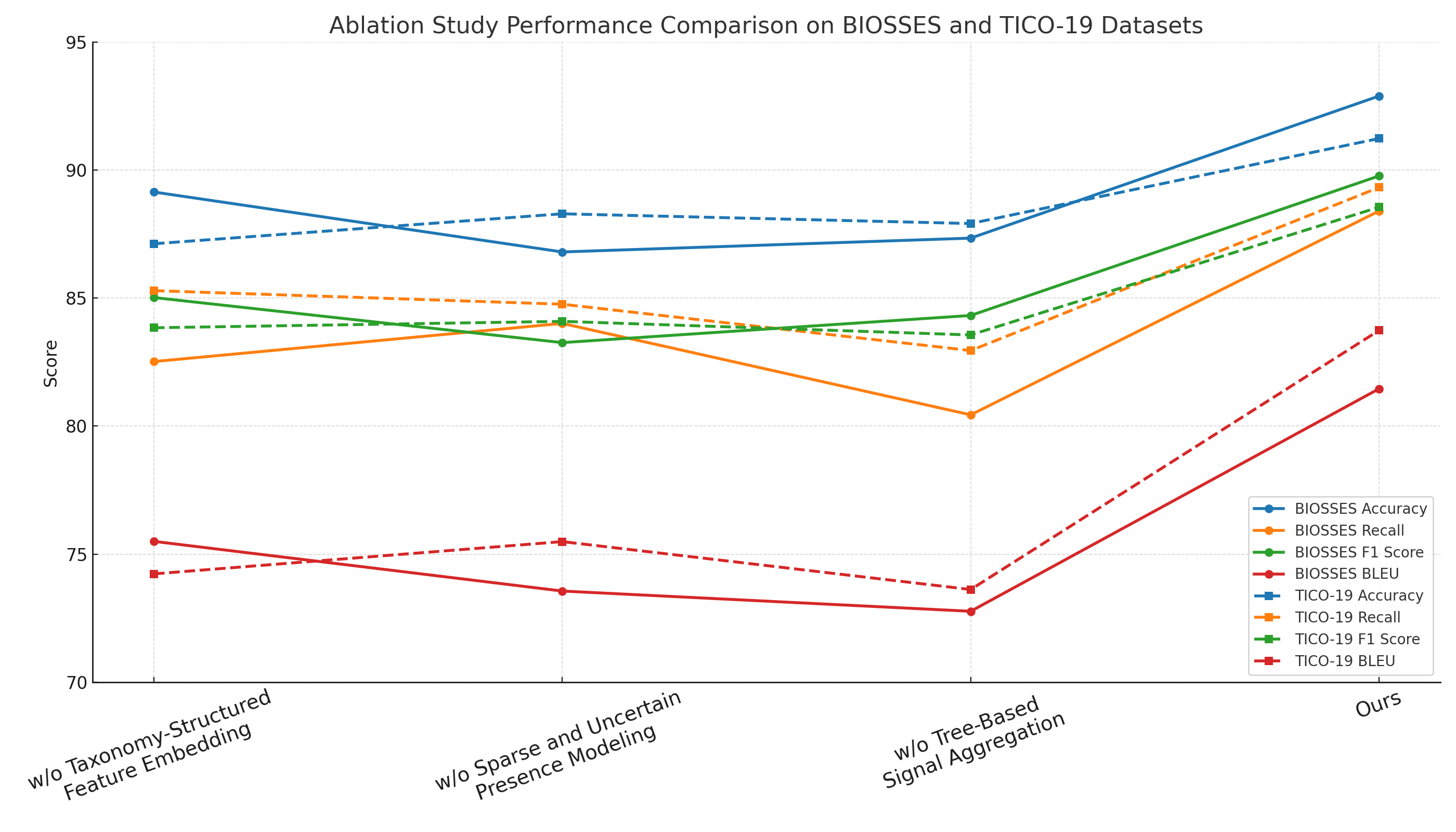

Supplement: Supplementary file 5 [file Image_1.png]

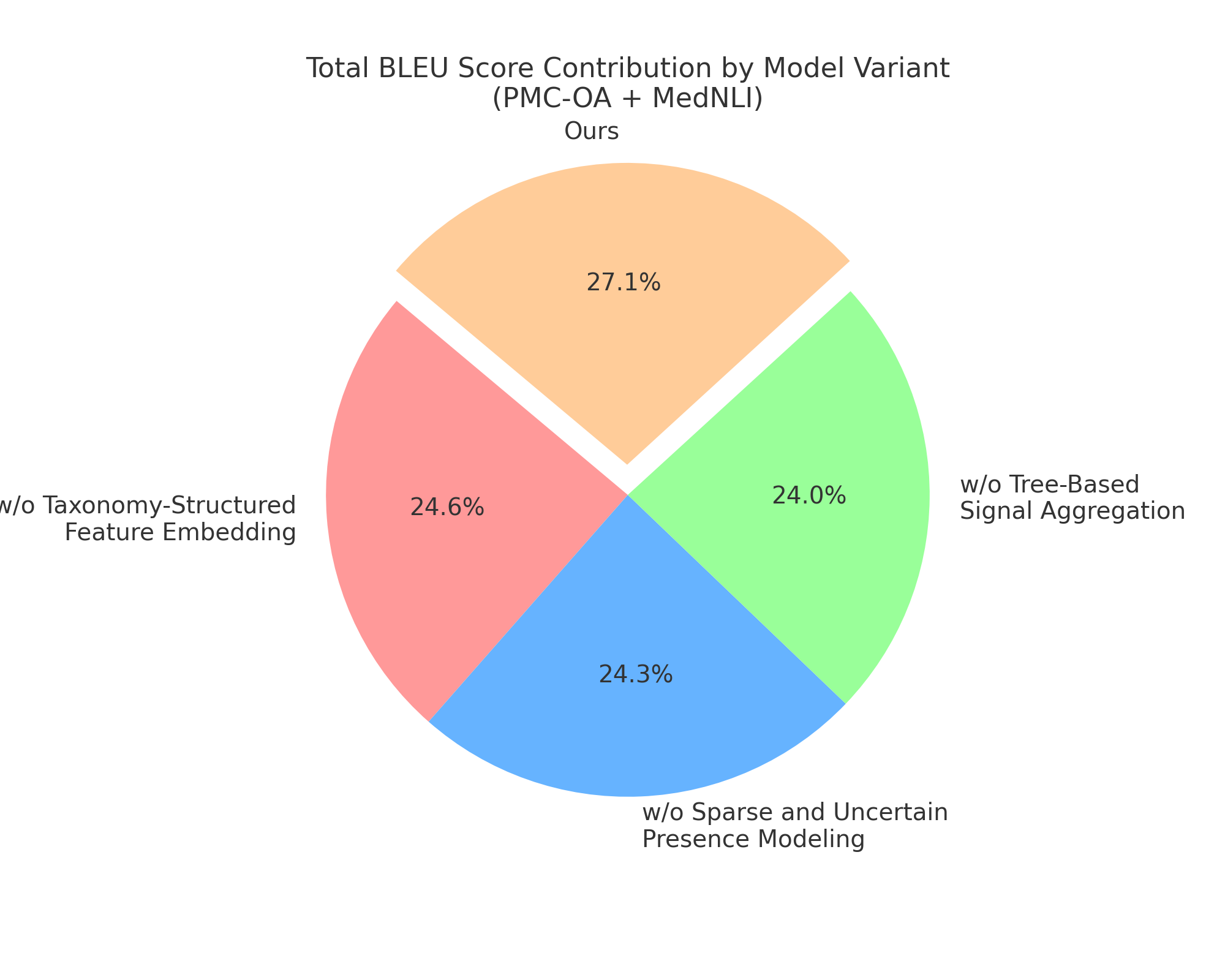

Supplement: Supplementary file 6 [file Image_2.png]

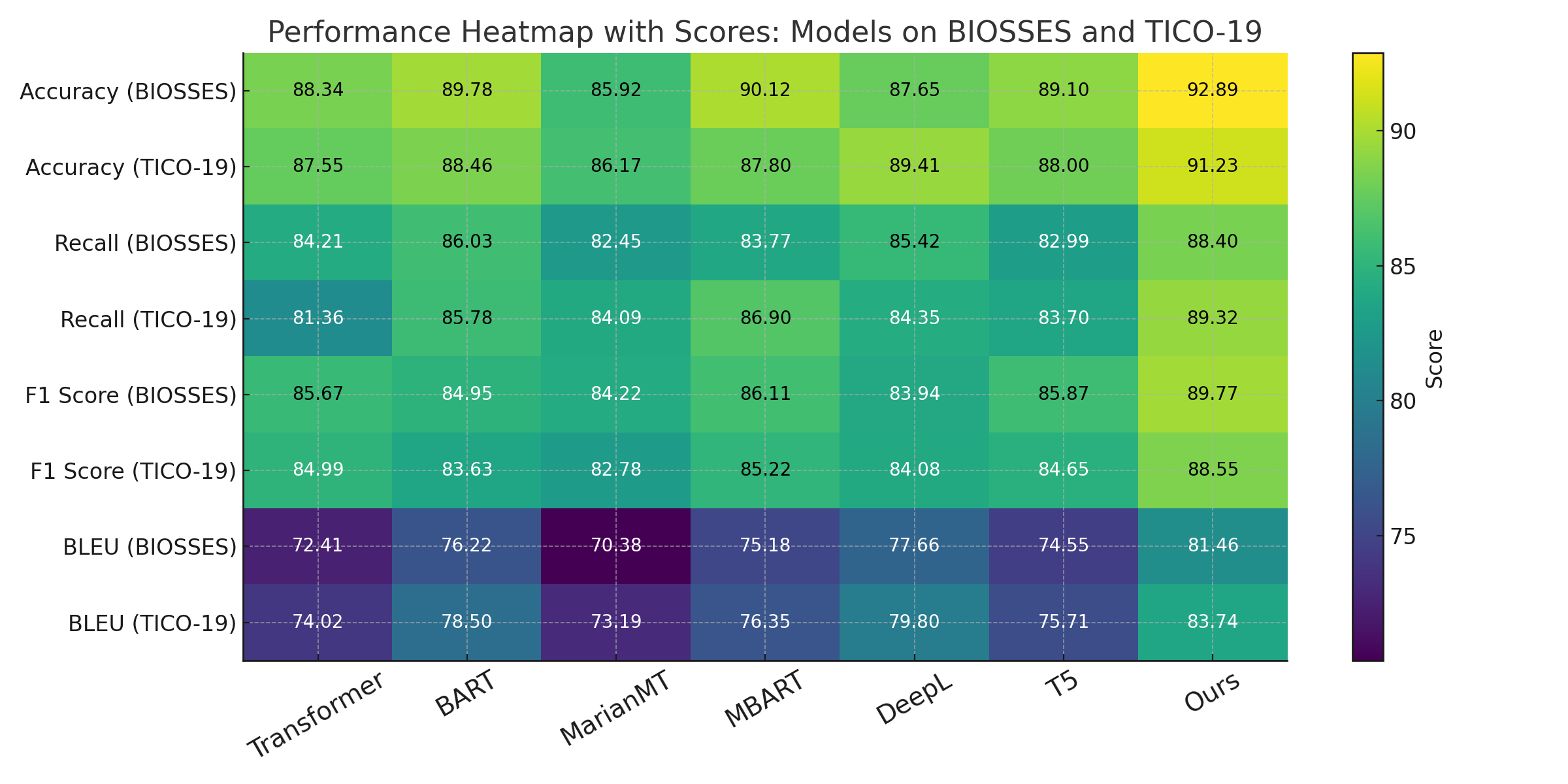

Supplement: Supplementary file 7 [file Image_3.png]

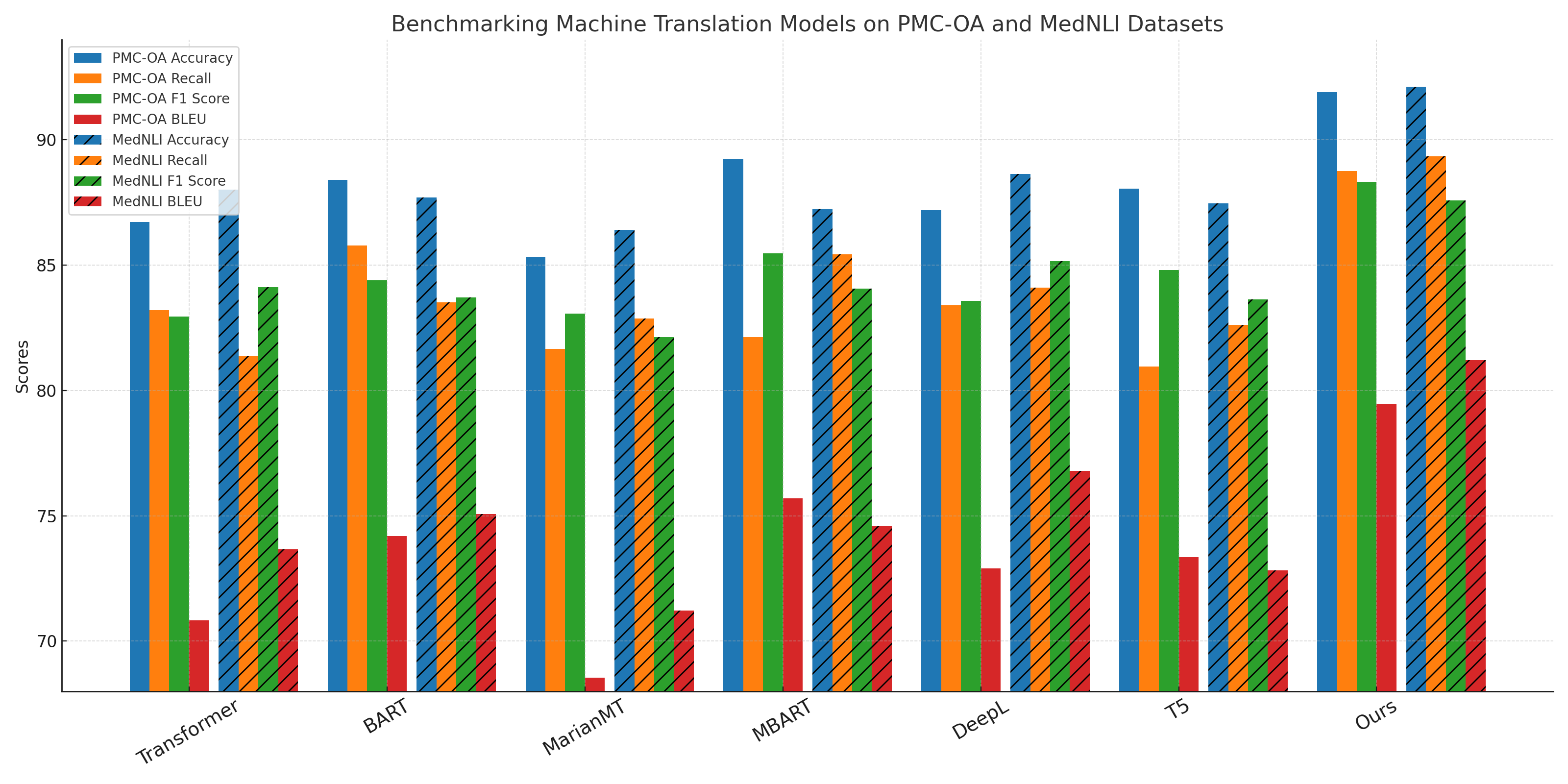

Supplement: Supplementary file 8 [file Image_4.png]
