# Supplementary material for: Enhancing pathogen identification through AI-assisted metagenomic sequencing
Source: Front Microbiol. 2025 Sep 19;16:1634194. doi: 10.3389/fmicb.2025.1634194 (PMC12493982; doi:10.3389/fmicb.2025.1634194)
Supplement: Supplementary file 4 [file Data_Sheet_4.pdf]

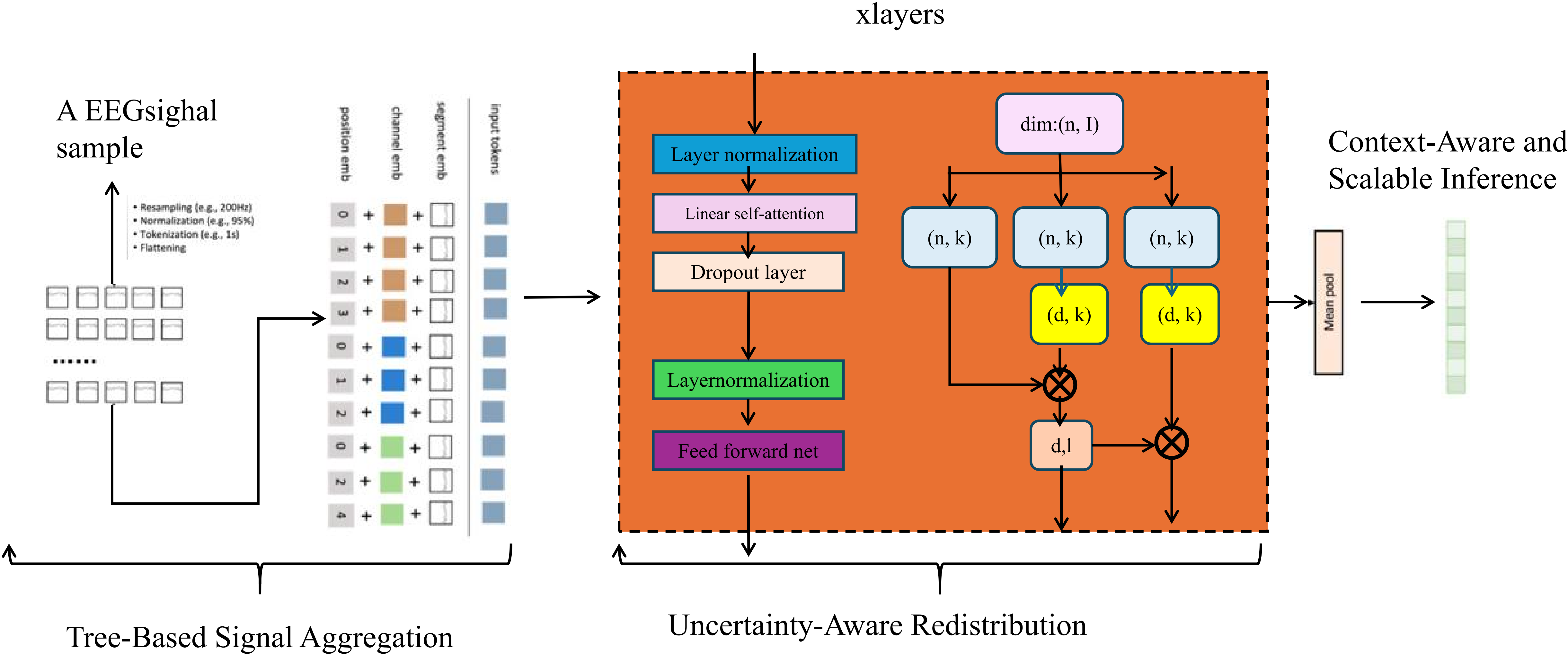
xlayers
A EEGsighal sample
• Resampling (e.g., 200Hz)
• Normalization (e.g., 95%)
• Tokenization (e.g., 1s)
• Flattening
position emb
channel emb
segment emb
input tokens
Layer normalization
Linear self-attention
Dropout layer
Layernormalization
Feed forward net
dim:(n, I)
(n, k)
(n, k)
(n, k)
(d, k)
(d, k)
d,l
Mean pool
Context-Aware and Scalable Inference
Tree-Based Signal Aggregation
Uncertainty-Aware Redistribution
